# Liquid Crystal Biosensors: Principles, Structure and Applications

**DOI:** 10.3390/bios12080639

**Published:** 2022-08-14

**Authors:** Haonan Wang, Tianhua Xu, Yaoxin Fu, Ziyihui Wang, Mark S. Leeson, Junfeng Jiang, Tiegen Liu

**Affiliations:** 1 School of Precision Instruments and Opto-Electronics Engineering, Tianjin University, Tianjin 300072, China; 2 School of Engineering, University of Warwick, Coventry CV4 7AL, UK; 3 School of Electrical and Electronics Engineering, Nanyang Technological University, Singapore 639798, Singapore

**Keywords:** liquid crystals, LC-based biosensors, microfluidics, whispering gallery mode, optofluidic

## Abstract

Liquid crystals (LCs) have been widely used as sensitive elements to construct LC biosensors based on the principle that specific bonding events between biomolecules can affect the orientation of LC molecules. On the basis of the sensing interface of LC molecules, LC biosensors can be classified into three types: LC–solid interface sensing platforms, LC–aqueous interface sensing platforms, and LC–droplet interface sensing platforms. In addition, as a signal amplification method, the combination of LCs and whispering gallery mode (WGM) optical microcavities can provide higher detection sensitivity due to the extremely high quality factor and the small mode volume of the WGM optical microcavity, which enhances the interaction between the light field and biotargets. In this review, we present an overview of the basic principles, the structure, and the applications of LC biosensors. We discuss the important properties of LC and the principle of LC biosensors. The different geometries of LCs in the biosensing systems as well as their applications in the biological detection are then described. The fabrication and the application of the LC-based WGM microcavity optofluidic sensor in the biological detection are also introduced. Finally, challenges and potential research opportunities in the development of LC-based biosensors are discussed.

## 1. Introduction

Liquid crystals (LCs) are phase transition materials that exist between the liquid and the crystal states, and they can flow as liquids and also have properties such as birefringence of crystals [1]. LCs can respond readily to external stimuli such as electromagnetic fields [2,3,4,5,6], pressure [7,8,9], surface effects [10,11,12,13], optical properties [14,15], temperature [16,17,18], and chemical analytes [19,20]. Changes in these factors can be monitored by various characterization techniques. Since LCs have superiorities in sensitivity, reactivity, and fabrications, research works on LCs have been expanded widely to address current technological and scientific challenges [21,22]. Accordingly, LC-based sensing techniques have gradually attracted more and more research attention.

LC molecules can be applied to serve as sensitive materials to respond to environmental changes or the occurrence of binding events. LCs generate macroscopic signals by magnifying and transforming molecular events in their surroundings [23]. The response properties of LCs have been extensively investigated in the context of a variety of sensing applications based on the molecular orientation. These properties form the foundation of LC-based sensing platforms [24,25,26,27,28,29]. A popular biosensing approach employs the optical birefringence properties of LC materials. Signal sensors can detect changes in the phases and the polarization states, since the refractive indices (ordinary and extraordinary refractive indices) vary with the direction of the light beam propagating along LCs. Interactions between biological receptors and ligands change the orientation of LC molecules, and this mechanism allows LCs to respond to biological or chemical materials [30,31,32,33,34]. In addition, LC molecules on the surface can transmit variations in their orientations within a vicinity (maximum 100 μm) to amplify the sensing signal [35]. Considering the optical birefringence properties and the high sensitivity to surface–interface interactions, LC biosensors can avoid the drawbacks of traditional biosensors, such as complex operations and the need for labeling biotargets [36]. Consequently, signals generated by LCs can be used to report subtle molecular events and external stimuli, and a number of materials-based optoelectronic signal transduction systems have been developed for LC-based biosensors [37,38,39].

In response to the rapid advancements of the microfluidic technology, optofluidic systems that combine optics and microfluidic devices have steadily demonstrated their distinct merits and potentials. The microfluidic technique is very important in the biomedical detection since it enables all the functions, including the preparation of biological samples [40], the measurement of concentrations [41,42], the monitoring of biochemical reactions [43,44,45,46], and the detection of products [47], to be implemented on a single biochip. Due to the vital role of the optical technologies in the biomedical detection, the integration of sample analysis, detection, and data transfer significantly enhances the efficiency. Moreover, fluids are excellent carriers of the light and matters, meaning that liquids with varying optical properties can be employed to manipulate the light and convey matters [48]. LCs have both optical anisotropy and liquid shapes, and this makes them perfectly suited for detecting and analyzing extremely small samples in biomedicine and chemistry.

This review emphasizes primarily three types of biosensing interfaces for LC biosensors: LC–solid interface, LC–aqueous interface, and LC–droplet interface. The first two are further divided into conventional sensors built on glass substrates and biosensors fabricated using microfluidic techniques, and several biosensing applications are mentioned. The LC–droplet interface is divided into droplets produced by stirring or ultrasonic emulsification and the droplets produced by the microfluidic technique. The fabrication of the LC-based whispering gallery mode (WGM) biosensor and its specialized detection application in the biological area are discussed in detail. Finally, the future development and challenges of the LC biosensing techniques are summarized.

## 2. Principle of LC-Based Biosensors

**Figure 1:**
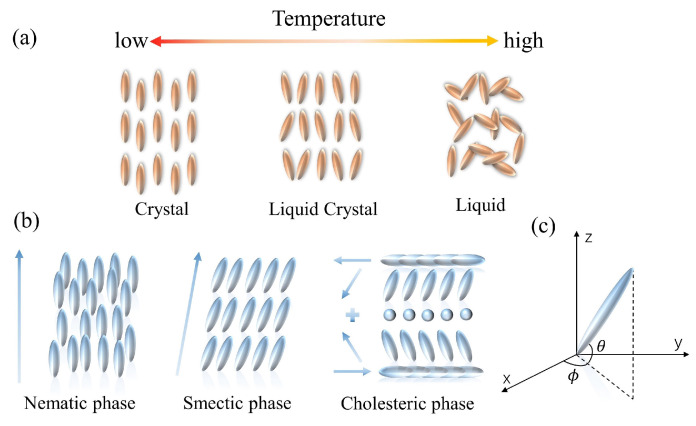
Schematic of the properties of LC molecules. (**a**) The arrangement of thermotropic LC molecules. (**b**) The variation of thermotropic LC molecules with temperature. (**c**) Illustration of LC directors in the Cartesian coordinate.

The mesophase of LCs is defined by the difference in conditions that create it. LCs can be categorized into two groups: lyotropic liquid crystals (LLCs) and thermotropic liquid crystals (TLCs). LLCs are composed of solvents and amphiphilic compounds. The phase transition of the LLCs depends on both the concentration and the temperature. The amphiphilic feature of one end being hydrophilic and the other end being hydrophobic is required for the lyotropic LCs, and the fabrication is dependent on the interactions between the amphiphilic molecules [49]. In contrast, the TLCs can only develop a liquid crystal phase within a limited temperature range (Figure 1a). Such LCs take on a more turbid appearance at the melting point. They continue to heat up until the clearing point is reached, at which point the LCs become transparent. According to the arrangement of the LC molecules, thermotropic LCs can be divided into three types: nematic liquid crystals (NLCs), cholesteric liquid crystals (CLCs), and smectic liquid crystals (SLCs) (Figure 1b). NLC molecules possess low van der Waals forces, excellent fluidity, and optical birefringence. Due to the high sensitivity to the environmental changes, NLC materials are widely investigated [50]. CLCs, also known as chiral NLC phases, are the chiral variants of orientationally ordered NLC phases. CLC molecules have a director that is periodically rotated along the perpendicular axis; the orientation rotates periodically about a vertical axis, generating a helical superstructure. The CLC possesses exceptional optical properties, including optical rotation, circularly polarized light dichroism, and selective light scattering [51]. An SLC molecule is rod- or strip-shaped. Molecules in SLCs tend to align in layers, and the layers stack on each other. It is noted that all LCs discussed in this review are thermotropic.

### 2.1. Optical Anisotropy

LC molecules exhibit the anisotropy in terms of refractive index, dielectric properties, elastic coefficient, etc., due to the structural peculiarities of LCs (between crystals and liquids) [52,53,54,55]. The anisotropy in the refractive index is the most commonly used optical birefringence characteristic. Plane polarized light is decomposed into two linearly polarized rays with different propagation rates, which are called ordinary ($n_{o}$) and extraordinary ($n_{e}$) rays, respectively [56]. The birefringence property of LC can be described as the difference between the refractive indices of $n_{o}$ and $n_{e}$. This phenomenon will cause a phase change as the light beam outputs from the LC medium. The phase difference is known as the optical retardation δ, and is given by [57]:

(1) $$\delta =\frac{2\pi d}{\lambda }\left(n_{e}-n_{o}\right)$$

where λ is the wavelength of the light and *d* is the thickness of the medium. Therefore, LC systems are able to regulate the polarization of light due to the birefringence and convert this into an optical image visible to the naked eye, which can be viewed using a polarized optical microscope (POM).

The birefringence on the surface of LC molecules can cause changes in the optical image, and this means that the monitoring of changes in the optical signal image using a POM allows the indirect detection of the variations in the alignment of LC molecules. Due to the interference of two orthogonal rays, the optical texture of LCs is visible through crossed polarizers. The linearly polarized light beam cannot get through in the case of the homeotropic alignment of the LC molecules, and this will result in dark optical images. The director configuration of LCs restricted inside particular geometries can be identified using the POM, by observing patterns produced by the interaction between the polarized light and LCs [58]. The transmitted light has the following intensity when LCs are sandwiched between crossed polarizers:

(2) $$I=I_{0}\sin^{2}2\varphi \sin^{2}\frac{\delta }{2}$$

where $I_{0}$ is the initial intensity of the light and φ is the angle of the LC orientations relative to the polarizer. This formula provides a good explanation of the fact that LC molecules can produce varied optical images when observed under the polarized light [59]. When LCs are positioned between two crossed polarizers, the intensity of the transmitted light *I* = 0 for the homeotropic alignment of LCs (δ = 0), and a dark optical image is observed. When the LC orientations are disturbed, δ≠0, I≠0, a bright image will be observed.

### 2.2. Orientations of LCs

Owing to the interaction force between LC molecules and hydrogen bonding, the long axes of LCs tend to align parallel to each other. In addition, the orientation of molecules follows a certain order, and the preferred direction is called the director of phase, which is the average direction that molecules point [60]. The unit vector $\vec{n}$ is used to describe the direction of the preferred orientation, and the spatial orientation of the director is normally specified by the polar angle θ and the azimuth angle φ, as shown in Figure 1c. In general, it can be used to characterize the macroscopic structure and the condition of LCs, as it describes the arrangement direction of LC molecules in space. While LCs are in the dynamic motion, they are oriented along the director for a longer period. Accordingly, an average indicator is required to quantify the degree of LC ordering, i.e., the order parameter *S* [61], which can be defined as follows:

(3) $$S=\frac{1}{2}\left(3\cos^{2}\theta -1\right)$$

where θ is the angle between the long axis of LC molecules and the director. The long axis orientation of molecules in isotropic liquids is disordered, and thus *S* = 0. At the state of fully parallel LC molecules, we have *S* = 1. Consequently, the order parameter *S*, which is utilized to quantify the degree of orientation of molecules with respect to the director, is an important physical property for LCs. However, *S* is temperature-dependent and will decrease with the increase of the temperature, because LCs will vary from an anisotropic crystalline state to an isotropic liquid state accordingly [11]. The LC director is essential in producing LC biosensors since the spatial orientation significantly affects the characteristics of LCs. As a result of the contact force between LC molecules, an ordered arrangement can be built to realize the function of sensing, and the orientations of LCs will be readjusted by external variables.

## 3. Geometries of LCs in Biosensing

Based on the sensing interfaces, LC biosensors can be classified into three categories: LC–solid interface [62], LC–aqueous interface [63], and LC–droplet interface [20]. For the three major geometries of LC biosensors, each has its own special advantages in the detection of biomolecules. LC–solid interface is simple in design, and is capable of realizing array structures. The LC–aqueous interface can be fully in contact with the biological sample to provide high mobility and feasibility for biotargets. Further improvement of the sensitivity can be achieved by combining LC droplets with the laser spectroscopy. Moreover, the microfluidic technology can also be applied to the fabrication of LC biosensors with different geometries in order to reduce the errors in the manual operation, to accurately determine sensing parameters and to improve the sensitivity.

### 3.1. LC–Solid Interface

#### 3.1.1. LC–Solid Interface on Glass Substrates

LC sensing techniques have gained attention gradually with the development in the integration of biology, optics, and materials. Abbot et al. developed the first LC sensor to enable the detection of avidin and anti-Bi-IgG in 1998 via a gold layer on the substrate and binding of ligands and receptors [64]. With the LC biosensor, the material does not need to be marked for the detection, and complex analytical instruments are not required. In the subsequent research, the group enhanced the fabrication process of the sensing substrate. By rubbing the self-assembled film of bovine serum albumin (BSA) on the surface of the glass substrate, the orientation of LC molecules followed the rubbing direction due to the generated anisotropy. This resulted in the application of an LC biosensor for the detection of anti-BSA IgG [65]. Using the anchor transition of LCs, Choi et al. designed an optical system to detect the binding events between avidin ligands and biotin receptors [66]. Schwartz et al. modified the LC–aqueous interface with octadecyltrimethylammonium bromide (OTAB) to construct a DNA-responsive LC sensing platform [67]. In 2009, Bi et al. developed a pH sensor based on LCs to implement the real-time monitoring of penicillin G hydrolysis by the surface-immobilized penicillinase [68]. Yang et al. realized an LC biosensor for the sensitive detection of heavy metal ions in 2013 [69]. Guo et al. established an LC sensing platform based on gold nanoparticles (AuNPs) to enhance the signal by doping 4-Cyano-4-pentylbiphenyl, nematic LC (5CB) with nickel nanospheres (NiNS) [14]. A wide range of biosensing applications have been developed using LC materials. The LC biosensing technology has merits of low power consumption, rapid response speed, visible signals to the naked eye, and easy miniaturization for array sensors. LCs offer potential applications for the label-free biosensing detection.

In the biodetection using such LC biosensors, POM is popularly utilized as the visual inspection approach to monitor the orientation transition of LC molecules. The orientation of LC molecules can be changed by the addition of biomolecules and the variation can be observed using crossed polarizers. To identify the target molecules, corresponding ligands (such as the immunoreaction or the enzymatic reaction) are added to the alignment reagent layer of the glass substrates without affecting the homeotropic alignment of LCs. This will result in a black pattern under POM [70,71].

**Figure 2:**
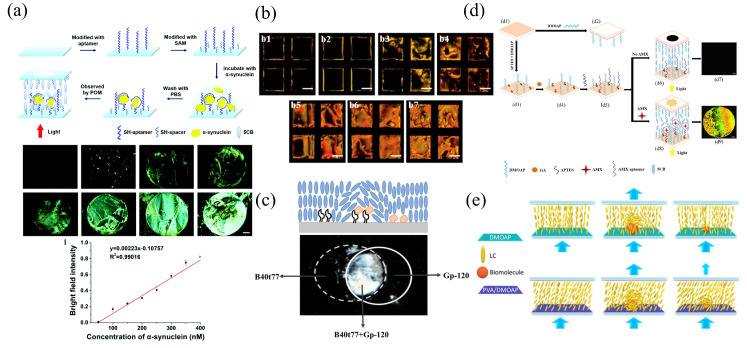
(**a**) Illustrations of the LC biosensor for the detection of α-syn and optical images of LC cells at different concentrations of α-syn [72]; reproduced with permission from Royal Society of Chemistry. (**b**) POM images of LC aptasensor cells with tetracycline at various concentrations of: (b1) 0.1 pM; (b2) 0.5 pM; (b3) 5 pM; (b4) 10 pM; (b5) 100 pM; (b6) 250 pM; (b7) 500 pM [74]; reproduced with permission from Elsevier. (**c**) POM images of the LC aptamer sensor [75]; copyright with permission from Abbasi et al. (**d**) Schematic illustration of the LC sensing strategy for detecting AMX (d1) Cleaned the bottom glass slide; (d2) upper glass slide; (d3) build self-assembled monolayer; (d4) grafting of GA onto APTES/DMOAP-modified glass substrate; (d5) immobilization of the AMX aptamer; (d6) absence of AMX results in LCs orienting homeotropically; (d7) POM image of the LC cell in the absence of AMX; (d8) binding of AMX to AMX aptamers disrupts the orientation of LCs; (d9) POM image of the LC cell in the presence of AMX [77]; copyright with permission from Nguyen et al. (**e**) Illustration of the DMOAP-coated and the PVA/DMOAP-coated substrates used to achieve the tilted state of the LCs [78]; copyright with permission from Chang et al.

In the biosensing, a branch of nucleic acid molecules known as aptamers are often employed as recognition components in biosensors due to their similar or superior characteristics to classical monoclonal antibodies. Yang et al. developed a DNA aptamer-based LC–solid interface biosensor for the detection of alpha-synuclein (α-syn), i.e., a protein associated with Parkinson’s disease (PD) [72], as shown in Figure 2a. The experiment made use of the F5R1 aptamer discovered by Zheng’s group [73]. The LC cell consisted of two specially functionalized glass slices, a cavity polyester film, and three dovetail clips. The capillary action was employed to inject the LCs into the LC cell cavity, which was then observed under the POM. The optical images were used to determine the biomolecule concentration. By varying the dosage of α-syn, it was discovered that α-syn was able to be bound precisely to the aptamer. With the increase of the concentration, the optical image under POM became brighter, and the intensity of the bright field in the image grew linearly with concentration. Zeinab et al. developed an LC biosensor to detect the tetracycline using aptamers and to estimate the quantity of the tetracycline by calculating the average grayscale intensity of POM images [74], as shown in Figure 2b. To ensure the homeotropic alignment of LCs while maintaining the darkness of the optical image, the optimal concentrations of APTES/DMOAP, GA, and aptamers have to be determined. The final detection limit for tetracycline was 0.5 pM. Abbasi et al. proposed a technique to identify HIV-1 glycoprotein-120 (gp-120) utilizing the B40t77 aptamers [75], with the aptamer screened by Dey et al. in 2005 [76], as shown in Figure 2c. It was found that the ideal concentration of B40t77 aptamer was 8 g per mL, and a 60-min fixation time was required. Otherwise, the aptamer would be incorrectly fixed, resulting in a diminished signal. Jang et al. reported a ligand-based LC biosensor for the detection of amoxicillin (AMX) [77], which was used in conjunction with the atomic force microscopy (AFM), to investigate the topographic characteristics of the surface substrate. Due to the aptamer specifically bound to the AMX molecule, a conformational shift occured in the aptamer, which disrupted the LC homeotropic orientation induced by the mixed self-assembled film of the APTES/DMOAP modified glass substrate. Bright optical images were obtained due to the disordered orientation of LC molecules with a final detection limit of 3.5 nM, as shown in Figure 2d. Sensor platforms based on the LC–solid interface using the glass substrates are simple to construct, stable to operate, and resistant to external interferences.

The detection limit against biomolecules can also be improved in the original LC biosensor through the technological or the chemical methods. A surface substrate immobilized with gold nanoparticles can enhance the disruption of LCs, and this can raise the detection limit for BSA by two orders of magnitude compared to the absence of gold nanoparticles [70,79]. In addition, the detection limit can be improved by varying the orientation of the LCs during the initialization. Changes in the grafting density of azobenzene-modified polymers on the glass surface can be used to introduce the planar anchoring of LCs. Kuang et al. achieved the planar anchoring of LCs by changing the grafting density of polymers modified with azobenzene on the glass surface. Azobenzene LC polymer-grafted gold nanoparticles are homeotropically aligned and the transition of the alignment (from homeotropic to random) can be efficiently achieved by the illumination [80]. Chang et al. developed a signal amplification approach in an LC biosensor [78] (Figure 2e) by employing PVA to preserve a small oblique alignment of LCs. Polyvinyl alcohol (PVA), a negatively charged planar alignment agent, which is able to introduce tiny pretilt angles, can be used with positively charged vertical surfactant DMOAP to produce a hybrid alignment layer on glass substrates, to adjust the tilted alignment state of LC molecules via the electrostatic interaction. This can increase the sensitivity of the LC sensor to detect biomolecules. The contact angle measurement was applied to evaluate the water contact angle on DMOAP-, PVA-, and PVA/DMOAP-modified glass substrates. Compared to the less hydrophilic DMOAP monolayer (contact angle of 84°) and the superhydrophilic PVA (contact angle of 2°), the contact angle decreases as the proportion of the PVA increases in the PVA/DMOAP hybrid layer. This thereby enhances the hydrophilicity of the glass surface and forms a uniform mixture coating. In addition, the average tilt angle on the PVA/DMOAP-coated glass substrate was evaluated using the capacitance measurement. In the absence of PVA, the tilt angle was around 85.85°, indicating that LCs were aligned homeopathically. With the increase of the PVA concentration, the average tilt angle decreased gradually. Therefore, by varying the proportion of the PVA in the PVA/DMOAP aligned composite, the tilt angle of LCs can be adjusted to find the optimal tilt angle to amplify the optical signal. Two nematic LCs, E7 and 5CB, were employed to quantify the BSA and the cortisol, via POM images. It was seen that E7 was more sensitive to changes in LCs than 5CB, which had a narrower nematic temperature and was prone to the phase transition at the ambient temperature. Through optical images under the POM, 2.5 × $10^{-8}$ μg ${\mathrm{mL}}^{-1}$ of BSA and 3 × $10^{-6}$ μg ${\mathrm{mL}}^{-1}$ of cortisol were detected. Both values were significantly lower than those obtained in the alignment layer using DMOAP only. This indicated a signal amplification by more than six orders of magnitude (10 μg ${\mathrm{mL}}^{-1}$ BSA and 0.1 μg ${\mathrm{mL}}^{-1}$ cortisol) [81,82], and demonstrated that the edge-tilted condition of the LC biosensing caused by the PVA/DMOAP aligned composite can significantly improve the optical response.

#### 3.1.2. Applications of Microfluidics at LC–Solid Interface

As a miniaturized fluid processing device, a microfluidic chip with microchannels ranging from tens to hundreds of microns can well control a small volume of fluid. Microfluidic systems have the advantages of high throughput, fewer samples, low loss, and high performance, and this makes them an attractive alternative to conventional biochemical reactions, which would save a lot of time and space [37]. With the rapid advancements of LC flow dynamics, microfabrication, and optical technologies, LCs have become a sub-component of microfluidic devices by establishing anchor directions on microchannels and controlling flow rates. By investigating processing techniques and materials of microfluidic chips and modifying surfaces with biochemical procedures, a microfluidic platform for detecting biochemical compounds was developed [83,84]. Currently, the soft lithography is the predominant technique to fabricate microfluidic chips. Employing polymers, such as polydimethylsiloxane (PDMS), microstructures can be produced using stamps with minute surface patterns [85].

The soft lithography relies on the photolithography and the molding of organic polymers to produce the elastomeric stamp rapidly and effectively. The photolithography technique is used to form a microstructure on the silicon wafer before embedding LC-fixed sensing interfaces into microfluidic channels. Exposed to the UV light and using a photomask, the photochemistry cures the photoresist. Photoresist is a light-sensitive liquid mixture that can transfer the required fine pattern from the mask to the substrate for processing via photochemical reaction, exposure, development, and other photolithography techniques. Organic polymer molding techniques include micro-molding in capillaries (MIMIC), micro-transfer molding (μTM), and replica molding [86]. The primary function of the mold-based soft lithography technique is to produce microfluidic devices that are hermetically sealed [40,87]. The LC–solid interfaces within microfluidic channels can be established by applying specific recognition ligands to glass substrates (coated with self-assembled membranes and bound to the microchannels). It is also possible to bind modified substrate to the PDMS microchannels before injecting ligands through the pumping device. In general, the soft lithography and the PDMS molding provide a number of advantages, including rapid prototyping, ease of fabrication, and good economic efficiency. Furthermore, Quake et al. demonstrated PDMS microvalves with footprints as small as 6 μm × 6 μm, which had high resolution and were applicable to surfaces of various materials and chemicals [88,89]. Biosensors based on microchannel devices using the interaction of channel surfaces and LC molecules have shown promising applications [90].

**Figure 3:**
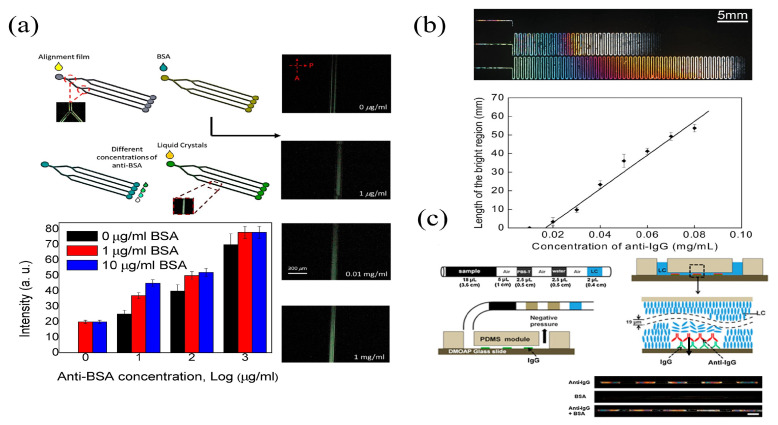
(**a**) Schematic of multi-microfluidic LC immunoassays with optical images of POM at different concentrations of BSA and intensities of LC microfluidic chips with BSA at concentrations of 0, 1, and 10 μg ${\mathrm{mL}}^{-1}$ [91]; reproduced with permission from Fan et al. (**b**) POM images of the IgG modified LC–solid interface microfluidic platform, and the linear characterization between the length of bright region and the IgG antibody concentration [92]; reproduced with permission from Wiley. (**c**) Diagram of the microfluidic LC sensor for automated immunoassays [93]; reproduced with permission from Elsevier.

Hsiao et al. developed an LC-based microfluidic device to detect the bovine serum albumin (BSA) [91]. The four microchannels were coated with DMOAP to initialize LC molecules with the homeotropic alignment, and the orientation of LC molecules was determined by the functions and geometric dimensions of the microchannels. Syringes were used to control the flow rate of LCs, BSA antigen, and BSA antibody solutions. It was found that the combination of the antigen and the antibody of BSA could provide a brighter optical appearance than the BSA-only case, as shown in Figure 3a. The presence of the BSA at 0.01 μg ${\mathrm{mL}}^{-1}$ and the BSA antibody at 1 μg ${\mathrm{mL}}^{-1}$ were determined. Combining the microfluidic technology and the LCs, Yang et al. established a label-free quantitative biosensing device to detect the concentration of antibody [92]. PDMS with embedded microchannels was hermetically bonded to a DMOAP-coated glass substrate, and the DMOAP modification induced a homeotropic arrangement of the LCs, which darkened the optical images. A planar alignment of LCs was introduced by the binding of anti-IgG to IgG, resulting in bright optical images in the microchannels. The size of the bright sections of LCs scaled with the concentration of anti-IgG. The detection limit for anti-IgG was 0.02 mg ${\mathrm{mL}}^{-1}$ in Figure 3b. Since the manual assembly and the liquid filling were required for microfluidic devices, Zhu et al. developed an integrated antigen–antibody detection system to eliminate manual errors [93]. The injection of the sample solution, the washing buffer, and the LCs into the tube was achieved via a syringe, which was connected to the flow input of the microfluidic device. The pressurization in the tube automated the immunoassay process. As the antigen was bound to the antibody on the microchannel wall, a dark-to-bright optical transition was visible under the POM, and the final detection limit of anti-rabbit IgG was 1 μg ${\mathrm{mL}}^{-1}$ in Figure 3c. In addition, the influence of the microchannel dimensions on the alignment of LCs was examined and the aspect ratio of microchannels was found to be critical. Therefore, the size of the microchannel has to be tailored before the fabrication of the LC–solid microfluidic interface in order to achieve the optimal performance. Microfluidic technologies enable the production of LC–solid interface-based sensors without the need for manual operations that can skew experimental results. The LC-based microfluidic chip can be applied to detect biomolecules as a revolutionary label-free biosensor.

### 3.2. LC–Aqueous Interface

#### 3.2.1. LC–Aqueous Interface on Glass Substrates

LC–aqueous biosensors are mainly implemented with functionalized glass slides, transmission electron microscopy (TEM) grids, LCs, and buffer solutions for sample analysis, where LCs are suspended within the TEM grids. The LC–aqueous interface facilitates the development of biosensors with higher sensitivity, while the aqueous interface has the advantages of preserving the function of biomolecules, providing high mobility and allowing a more comprehensive study of biochemical reactions in dynamic conditions. Since one interface in the LC–aqueous biosensor is in contact with the aqueous solution, it is required to introduce surfactants or other biologically self-assembled films to the aqueous interface to induce the homeotropic alignment of LCs. Currently, phospholipids and anionic and cationic surfactants are the most widely utilized surfactants [94,95,96]. Amphiphilic molecules will adhere to the stroma due to the presence of polar groups, and interactions between non-polar terminal alkyl chains and carbon chains will cause LC molecules to align homeotropically along the LC–aqueous interface. Due to the interaction between the target and the amphiphilic modifier molecules, the alignment of the LC molecules is disrupted. This results in the dark to bright transition of optical images, and thereby realize the detection of the target molecules [63].

**Figure 4:**
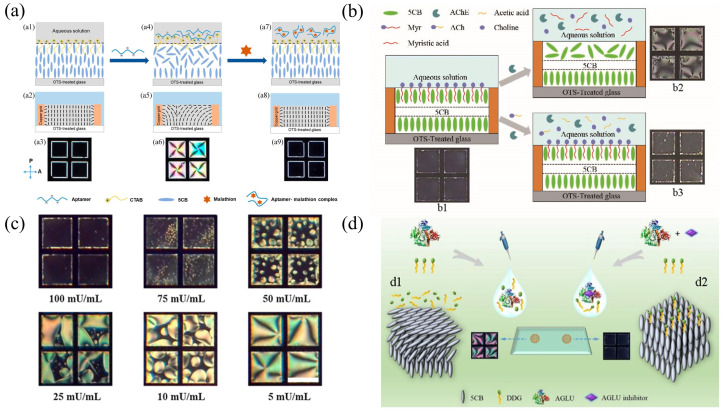
(**a**) Schematic of LC sensor device for malathion detection [97]; copyright with permission from Nguyen et al. (**b**) Diagram of the LC sensor configuration for the detection of acetylcholine (b1) Dark appearance associated with homeotropic orientation caused by Myr layer formation; (b2) enzymatic hydrolysis of Myr layer by AChE causes a bright appearance associated with a plane orientation; (b3) the competitive enzymatic hydrolysis of Myr and ACh by AChE is associated with homeotropic orientation and dark appearance [98]; reproduced with permission from Elsevier. (**c**) Optical images of LC sensor in the presence of various hydrogen peroxide concentrations [99]; reproduced with permission from Elsevier. (**d**) Schematic representation of LC-based AGLU analysis to detect antidiabetic drugs [100]; reproduced with permission from Elsevier.

Jang et al. developed a novel label-free LC biosensor to detect malathion insecticides [97]. DNA aptamers were used as sensing probes, specifically bound to malathion molecules, and the positively charged hexadecyltrimethylammonium bromide (CTAB) was chosen as the cationic surfactant. The CTAB was specifically bound to negatively charged DNA aptamers via electrostatic interactions. DNA aptamers can modify the structure of CTAB monolayers in the absence of malathion, resulting in the planar orientation of LCs at the interface and a dark-to-bright transition in the optical image. In the presence of malathion, DNA aptamers will preferentially interact with the malathion and release the CTAB due to the higher affinity. Afterwards, the free amphiphilic CTAB will self-assemble on the LC interface, and the optical image becomes dark, as shown in Figure 4a. The biosensor was successfully applied to detect the malathion in the river water, the tap water, and apple samples with high sensitivity (the sample recovery rate was 87.96–110.23%). Zhang et al. developed an LC–aqueous interface biosensor to detect acetylcholine (ACh) using myristylcholine (Myr) as the modified molecule [98]. A self-assembled monolayer film was realized at the LC–aqueous phase boundary to induce the homeotropic alignment of LC molecules. This led to dark optical images under the POM, as shown in Figure 4b. Since the acetylcholinesterase (AChE) solution was applied to the LC–aqueous interface, the enzymatic hydrolysis of Myr caused the orientation of LCs to vary from homeotropic to oblique at the interface, which thereby changed the optical images from dark to bright. After sequentially transferring the ACh and the AChE solutions to a Myr-modified LC interface, ACh occupied the enzymatic reaction site of AChE with a priority, which inhibited the hydrolysis. The Myr monolayer membrane at the interface can thus exist in a stable state. The orientation of LC molecules remained homeotropic and the optical image stayed dark. Such sensor had a simple operation and unlabeled characteristic, and its detection limit for ACh can reach 14.5 nM.

Along with the small molecules discussed above, LC–aqueous interface biosensors can support a lot of applications in the field of enzyme detection. Yu et al. investigated the interaction mechanism between a surfactant (PBEC14A) and hydrogen peroxide ($H_{2}O_{2}$) using an LC biosensor [99] and discovered that PBEC14A can be decomposed by $H_{2}O_{2}$. When PBEC14A is doped into LCs and was immersed in the aqueous solution, a self-assembled monolayer can be developed at the LC–aqueous interface to induce the homeotropic alignment of LC molecules. A dark polarized image can be observed under the POM, as shown in Figure 4c. $H_{2}O_{2}$ interacted with PBEC14A at the LC–aqueous interface, which led to the decomposition of PBEC14A. This destroyed the self-assembled monolayer and resulted in bright polarized images. With the coexistence of catalase and $H_{2}O_{2}$, the latter ($H_{2}O_{2}$) would be hydrolyzed by the former (catalase), and this would trigger a bright to dark change in the optical response of the LCs. The final detection limit for catalase was 5.5 mU ${\mathrm{mL}}^{-1}$, with relatively good practical detection in human serum samples (errors range from −6.8–9.4%). Sun et al. modified the LC–aqueous interface using a nonionic surfactant (DDG) as a self-assembled monolayer and built a α-glucosidase (AGLU) inhibitor-responsive LC sensing platform, as shown in Figure 4d [100]. Due to the enzymatic hydrolysis of AGLU, the monolayer formed by DDG was disrupted, resulting in an oblique alignment transition of LCs at the LC–aqueous interface, and a dark to bright transition in the optical images. The detection limits for the three antidiabetic medicines, acarbose, miglisol, and voglibose, were 0.57 μM, 1.00 μM, and 0.01 μM, respectively.

#### 3.2.2. Applications of Microfluidics at the LC–Aqueous Interface

The aforementioned conventional LC–aqueous interface in glass-substrate-based biosensors employs a manual approach to pipette LCs into TEM grids [97,98,99,100]. In spite of the simple design and the mature technology, the implementation of such conventional sensors still requires the manual operation, which is laborious and time-consuming. Hence, it is necessary to develop automated and reproducible methods to fabricate LC films without complicated physical manipulations. Microfluidic technologies have contributed significantly to the production of interfaces that mimic fluid biological membranes for the study of dynamic response processes.

**Figure 5:**
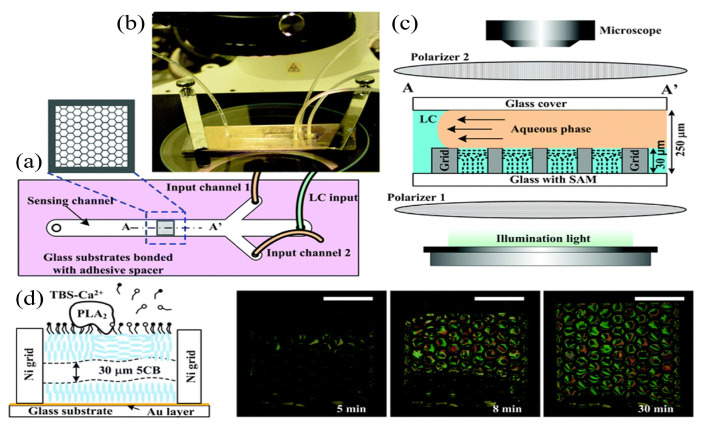
Setup of LC microfluidic biosensing system. (**a**) Top view diagram of the system, with hexagonal squares supporting the grid structure. (**b**) Physical picture of the system. (**c**) System was situated between two crossed polarizers with the shear force of the water to form the LC–aqueous interface. (**d**) The SAM was hydrolyzed in the presence of ${\mathrm{Ca}}^{2+}$, and the 5CB film in the central grid of the sensing channel was observed with transition (from dark to bright) in the optical image [102]; reproduced with permission from Royal Society of Chemistry.

To fabricate LC–aqueous interfaces within microchannels, the homeotropic orientation of LCs requires the excitation from surface-coated microchannel surfactants, and tightly bonds glass substrates with gate microstructures to PDMS microchannels [84] (Figure 5a,b). By pumping LCs, deionized water, and amphiphilic molecules into the microchannel, the shear force generated by the laminar flow of the deionized water can remove the excessive LCs above the microfluidic structure which can establish the LC–aqueous interface sensor [101].

Jiang et al. developed an LC–aqueous interface biosensor to monitor enzymatic processes and to identify phospholipases using microfluidics [102]. During the rupture of the self-assembled monolayer, the orientation of LCs at the LC–aqueous interface changed from homeotropic to planar, leading to the transition of optical images from dark to bright. L-α-dilauroylphosphatidylcholine (L-DLPC) molecules assembled in microchannels can be hydrolyzed by phospholipase $A_{2}$ in the presence of ${\mathrm{Ca}}^{2+}$. This resulted in the rearrangement of lipid molecules and the transition of LC orientations at the interface. A dark to bright transition was observed under the POM (Figure 5c,d). Enzymatic reactions could be monitored biochemically using the microfluidic device. Moreover, the interaction between LCs and the aqueous solution in the microchannel during the removal of excessive LCs was also investigated. With the increment of the flow rate of the aqueous solution, the pressure in the liquid film would also increase. This could result in the damage of instrument and the changes to the microstructure of the liquid film. The selection of the fluid parameters in the microchannel is therefore crucial to establish the LC–aqueous interface.

### 3.3. LC Droplets

#### 3.3.1. LC Droplet Biosensing Integrated with Spectroscopy

Recently, LC droplets have been extensively investigated. Due to the enormous surface-to-volume ratio and the fact that tiny changes in the surface anchoring energy can result in signal amplification and achieve a lower LOD, LC droplets have distinct advantages in the field of highly sensitive biosensors [103]. Typically, LC droplets can be fabricated via the stirring emulsification, the ultrasonication emulsification, the membrane emulsification, and the microfluidic encapsulation. When amphiphilic material molecules (such as the lipid) are added to the aqueous solution, with the increase of amphiphilic molecules adsorbed on the surface of LC droplets, LC molecules can be affected to produce a homeotropically aligned orientation. Thus, the application of LC droplets to the detection of bacteria was developed [104]. Jang et al. reported that the outer membranes of Gram-negative bacteria and enveloped viruses were composed of lipids, and the configuration of LC droplets changed from bipolar to radial after the contact with bacteria or viruses [105]. Abbott et al. demonstrated the use of LC droplets to detect the concentration of endotoxin, composed of the glycophospholipid (or lipid A) [106,107]. Bacterial endotoxin and its six-tailed glycolipid component lipid A can trigger the configurational transition of LC droplets from bipolar to radial at extremely low concentrations. Unlike common surfactants and lipids, which require a large amount of coating on the surface of LC droplets, the six-tailed synthetic amphiphilic molecules can generate inverted self-associative nanostructures at the topological defects of LC droplets. This thereby can trigger the orientation transition of LC molecules at lower endotoxin concentration. Compared to polymerase chain reaction (PCR) and flow cytometry, and other sophisticated and expensive cell detection techniques, LC droplets have provided a novel and cost-effective solution for the cell identification. The configuration transition of LC droplets can be used for the sensitive detection in the biological areas and has shown excellent application prospects.

**Figure 6:**
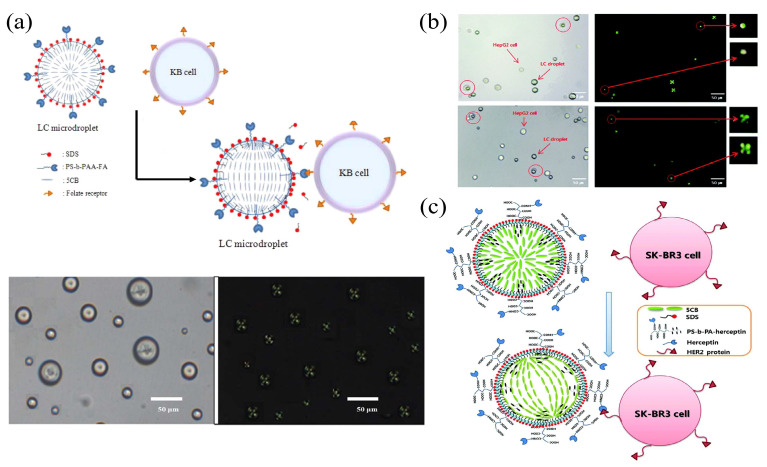
(**a**) Illustration of the configuration transition and POM images of LC droplets containing PS-b-PAA-FA with KB cancer cells [108]; reproduced with permission from American Chemical Society. (**b**) Optical and polarization images of HepG2 cells in contact with β-galactose-(PS-b-PA-G) anchored LC droplets [109]; reproduced with permission from Royal Society of Chemistry. (**c**) Illustration of herceptin–HER2-interaction-induced configuration transition in LC droplets [110]; reproduced with permission from Royal Society of Chemistry.

In the medical field, cancer is still a difficult problem that has not been fully resolved. The detection of cancer cell markers can be used to aid the early diagnosis and the treatment of cancer. Kang et al. anchored a folate-receptor-specific ligand (FA) on the PS-b-PAA-coated LC droplets to implement the specific detection of KB cancer cells [108]. The FA located on the surface of LC droplets is a specific ligand for the folate receptor of KB cancer cells. Interactions from the ligand–receptor binding triggered a transition from the radial to the bipolar configuration in LC droplets (Figure 6a). In addition, the β-galactose-conjugated poly (styrene-b-acrylic acid) block copolymer (PS-b-PA-G) and the sodium dodecyl sulfate (SDS) were utilized to modify LC droplets to detect the HepG2 cell [109]. The ligand–receptor interaction between HepG2 cells and the β-galactose-conjugated block copolymer can produce the transition (from radial to bipolar) in the orientation of LC molecules and is highly selective for HepG2 cells (Figure 6b). Ding et al. demonstrated that LC droplets can be applied to detect SK-BR3 human breast cancer cells [110], and that a poly (styrene-b-acrylic acid) amphiphilic block copolymer (PS-b-PA) can interact with overexpressed HER2+ on breast cancer cells, leading to the transition of the configuration (from radial to bipolar) in LC droplets (Figure 6c). With the advancements of molecular biology and biomedical detection techniques, an increasing number of cancer cell markers have been identified and analyzed. The use of LC droplets to detect cancer cells is rapid, visible to the naked eye, user-friendly, and highly effective. It has excellent specificity and can provide a novel platform for the early diagnosis of cancer.

Whispering gallery mode (WGM) optical microcavities have been widely investigated in the sensing field, due to the simple construction and the high Q factor. Due to the spherical and smooth surfaces of LC droplets, WGM microcavities can be employed for the biosensing by monitoring the wavelength or the mode of the laser emission, where the binding of biomolecules will change the orientation of LC molecules. Therefore, laser spectroscopy was applied to detect the concentration of biochemicals using a more efficient and precise approach. By combining the naked eye inspection under POM and the laser spectroscopy, a more sensitive and precise characterization can be achieved [111,112].

**Figure 7:**
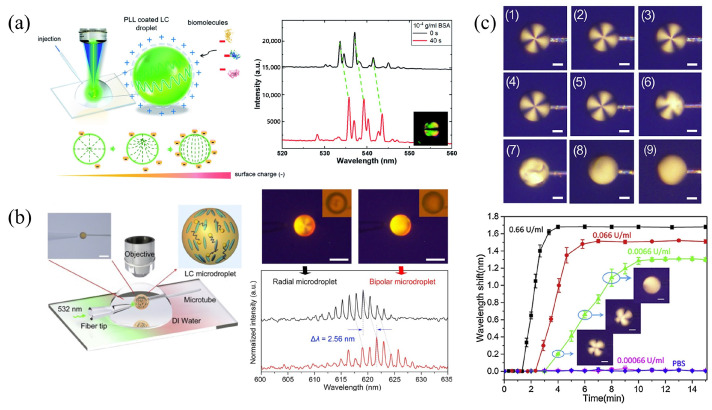
(**a**) Illustration of the experimental setup and the shift of the laser wavelength due to different concentrations of BSA [113]; reproduced with permission from Royal Society of Chemistry. (**b**) Schematic representation of the lasing experimental setup and wavelength shift of LC droplets with configuration transition from bipolar to radial [114]; reproduced with permission from Elsevier. (**c**) POM images of LC droplets in different concentrations of Myr and spectral responses at different concentrations of AChE [115]; reproduced with permission from Elsevier.

Bioelectrostatics play a profound role in the life processes of organisms. Wang et al. constructed a bioelectrostatically responsive microlaser and employed the laser wavelength shift as the sensing parameter to detect negatively charged biological molecules [113]. Due to electrostatic interactions between molecules, poly-L-lysine (PLL)-coated LC droplets have a positive charge, which can attract negatively charged biomolecules and result in the orientation transition of LC molecules. The detection capability of the microlaser was tested in practical applications using BSA as the biomolecule under test, as shown in Figure 7a. The wavelength shift in the whole laser spectrum, scaled proportional to the BSA concentration, and the ultimate detection limit was 0.36 pM ($2.4\times 10^{-11}$ g ${\mathrm{mL}}^{-1}$). Compared to conventional approaches, e.g., the POM detection ($10^{-7}$ g ${\mathrm{mL}}^{-1}$), a significant improvement in the sensitivity was achieved. Due to the combination of the WGM microcavity and LCs, the binding signal of the molecules was substantially amplified, and thus the detection of biomolecules at lower concentrations were enabled.

In addition, LC droplets acting as WGM resonators have a wide range of applications in the detection and the monitoring of enzymatic processes [59,60]. An LC droplet sensor based on the penicillinase reaction of pH changes was designed, where 5CB droplets were doped with 4′-pentyl-biphenyl-4-carboxylic acid (PBA) [114]. The carboxylic acid portion of PBA is pH-sensitive in aqueous solutions. When the pH of phosphate buffer solution (PBS) in a liquid environment is increased from 5.7 to 6.0, a (bipolar to radial) transition of LC molecules can be observed. With the pulsed pumping of a 532 nm laser, a red-shift of the laser spectrum was observed when the pH value decreased (Figure 7b). The LC droplet can be used to monitor the enzymatic reaction in penicillinase, based on the principle that the hydrolysis of penicillin G can generate H+ to reduce the pH value of the solution. As the concentration of penicillin G increased, the degree of the spectral red-shift was also enhanced. The final detection limit of the concentration of penicillin G was about 2 μM. In addition, LC droplets can be utilized to detect acetylcholinease (AChE) and its inhibitors in a real-time manner without the requirement of labeling [115]. In the presence of myristoylcholine chloride (Myr, the substrate of AChE), AChE can effectively catalyze the enzymatic hydrolysis of Myr, inducing the configuration transition of LC molecules. This results in a significant variation of the refractive index, as shown in Figure 7c. The TE mode corresponds to the transition of an LC droplet, from bipolar to radial, where the anomalous refractive index ($n_{e}$) of LC becomes normal ($n_{o}$). This also leads to a red-shift of the WGM laser spectrum. The detection range of acetylcholinesterase was from 0.0066 U ${\mathrm{mL}}^{-1}$ to 66 U ${\mathrm{mL}}^{-1}$.

In general, since subtle changes are difficult to observe directly by the naked eye or the microscope, the combination of the WGM microcavity and LCs can be employed to double-amplify the signal. The above works provide insights and experiences for the design and the development of laser-based LC biosensors and demonstrate the enormous potential for the development of optical sensing platforms in the biodetection. There are also possibilities for LC droplet biosensors to be further integrated into portable and low-cost sensing platforms.

#### 3.3.2. LC Droplet Microfluidic Biosensors

The size of LC droplets produced by the stirring and ultrasonication emulsification cannot be precisely controlled. Daniel et al. reported that the transition of the LC orientation induced by endotoxin (from bipolar to radial) highly depends on the size of LC droplets [116]. As the diameter of the LC droplet increases from 2 μm to more than 10 μm, the proportion of LCs, with configurations transiting from bipolar to radial, will decrease. Consequently, the size of the LC droplet poses a significant impact on the performance of the sensor. Droplet microfluidics can fabricate and manipulate droplets via microfluidic channels in a configurable manner, which enables the production of stable monodisperse droplets. This provides effective technical approaches to resolve challenging and complex problems in biology and chemistry [117,118].

**Figure 8:**
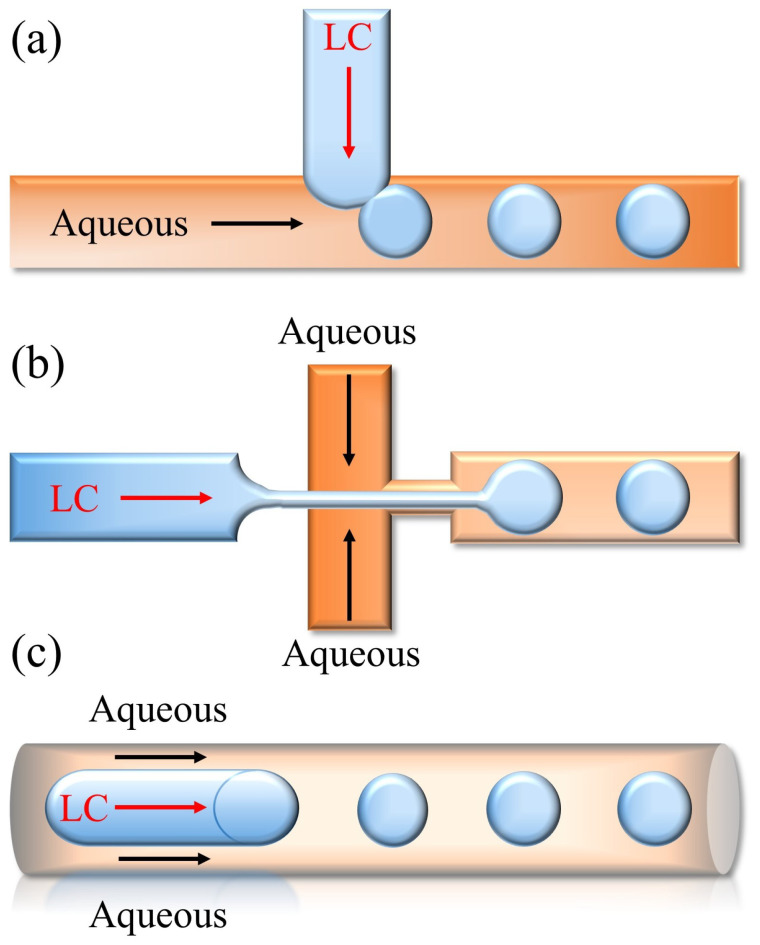
LC droplets generation strategies: (**a**) T-junction structure; (**b**) flow-focusing structure; (**c**) co-flowing structure.

Generally, droplets are formed by applying an external force to disrupt the equilibrium of the tension between the immiscible continuous phase and the dispersed phase. The T-junction structure, the flow-focusing structure, and the co-flowing structure are the most widely utilized microfluidic structures in producing monodisperse LC droplets. The generation of droplets by the T-junction was firstly proposed by Thorsen [119]. The LC and the aqueous phases flow through two perpendicular channels, and droplets are produced at the intersection of channels. LC droplets are the product from the combined action of the shear force and the surface tension between two phases, as shown in Figure 8a. The flow-focusing structure employs two crossed channels, and the aqueous phase exerts the symmetrical shearing action on the LC phase from both sides in order to produce LC droplets, which are more stable and convenient to control (Figure 8b). The co-flowing structure utilizes two coaxially nested microchannels, and the aqueous phase surrounds and shears the LC phase to produce LC droplets. The application of the co-flowing to obtain droplets can effectively prevent the cross-contamination between the dispersed phase in droplets and the channel wall, and the produced LC droplets are stable and have uniform sizes (Figure 8c). It should be noted that both the flow rate of two phases and the diameter of the microchannel can affect the size of the droplet. Cramer et al. examined the impact of the flow rate, the liquid viscosity, and the interfacial tension on the generation of confluent droplets [120]. Results showed that with the increment of the flow rates of the continuous phase (LC) and the dispersed phase (aqueous solution), the viscosity of the liquid, and the reduction of the interfacial tension, the tendency of the production of droplets increases. Therefore, the influence of these parameters should also be fully considered when producing LC droplets.

**Figure 9:**
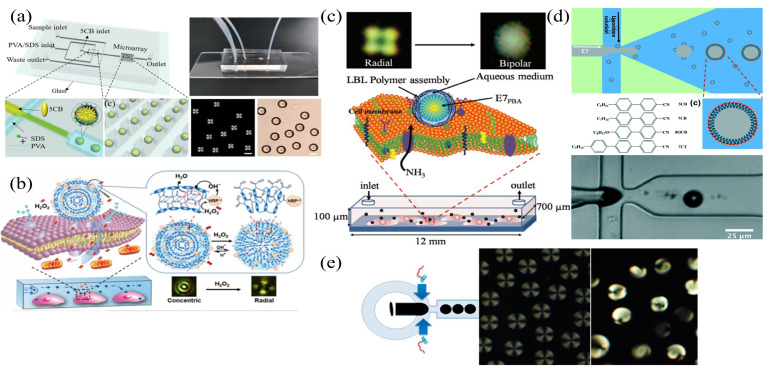
(**a**) Schematic of the LC droplet device for the determination of bile acids [121]; reproduced with permission from Elsevier. (**b**) Principle of configuration transformation of LCEM-HRP immobilized on the cell membrane based on $H_{2}O_{2}$ reduction reaction [123]; reproduced with permission from Wiley. (**c**) Diagram of $P-E7_{\mathrm{PBA}}$ immobilized on cultured cells in a microfluidic channel and POM images of the transition from radial to bipolar [124]; reproduced with permission from Wiley. (**d**) Production of lipid-coated LC droplets for the detection of SMP43 according to the principle of the configuration transition in LC droplets caused by the disruption of the lipid membrane layer [125]; reproduced with permission from Royal Society of Chemistry. (**e**) LC glucose sensors were prepared by covalently bonding GOx to PAA-b-LCP functionalized LC droplets with PAA chains, and the pH change induced by GOx-catalyzed glucose oxidation underwent a radial to bipolar configuration transition through a change in the PAA chains [126]; reproduced with permission from American Chemical Society.

The bile acid is the final product of the cholesterol metabolism in the human liver and it has a close relationship with cholesterol. Han et al. developed a flow-focusing microfluidic chip to detect bile acids using monodisperse LC droplets as optical probes, with LCs as the dispersed phase and amphiphilic sodium dodecyl sulfate (SDS) as the continuous phase [121,122] (Figure 9a). The adsorption of bile acid molecules on the surface of LC droplets can disrupt the alignment of SDS, and this results in a change in the orientation of LC molecules within the droplets. The transition of the LC droplet configuration, from radial to bipolar, was observed by POM. In addition, the impact of parameters, e.g., the two-phase flow rate, the continuous phase viscosity, and the orifice size on the production of LC droplets, were examined, similar to the influence of the droplet size on the detection performance. It was found that the detection limit of the bile acid dropped as the LC droplet size increased. This indicated that the LC droplet with a larger size was more likely to result in a configuration transition of the LC molecules. LC droplets produced by the microfluidic technology can be effectively utilized in the field of cell analysis. Li et al. developed an LC droplet biosensor for the real-time monitoring of $H_{2}O_{2}$ released in living cells [123] (Figure 9b). The real-time release of $H_{2}O_{2}$ in the individual living cell can be observed by directly immobilizing chemically reactive LC elastomer microspheres (LCEM-HRP) with horseradish peroxidase functionality on cell membranes. The modified horseradish peroxidase (HRP) on LCEM-HRP promoted the reduction of $H_{2}O_{2}$. This resulted in the deprotonation of microspheres and the breakdown of internal hydrogen bonds, and induced the change of LC molecules (from concentric to radial). In addition, a droplet-based biosensor in detecting ammonia (${\mathrm{NH}}_{3}$) was implemented, which played a crucial role in diagnosing tumor cells [124] (Figure 9c). $P-E7_{\mathrm{PBA}}$ was prepared by encapsulating 4-pentylbiphenyl-4′-carboxylic acid (PBA)-doped LC (E7) into polyelectrolyte self-assembled microcapsules. $P-E7_{\mathrm{PBA}}$ immobilized on the cell membrane can respond to ${\mathrm{NH}}_{3}$ released by the cell and can cause a change in the surface charge density, inducing a transition in the orientation of LC molecules (from radial to bipolar).

To identify tiny molecules, Bao et al. developed a lipid-modified surface LC droplet, using the flow-focusing microfluidic device, to detect Smp43, which is an antimicrobial peptide (AMPs) in scorpion venom [125] (Figure 9d). The interaction between Smp43 and lipid molecules on the surface results in the configuration transition of LC droplets (from radial to bipolar). This could be captured under POM, and Smp43 had a detection limit of 3.6 μM. Kim et al. employed microfluidics to fabricate LC droplets and coated 5CB droplets with pH-responsive polymer acrylic acid-b-4-cyclodiphenyl-4-oxoundecylacrylate (PAA-b-LCP). The immobilization of glucose oxidase (GOx) on PAA chains thus generated a biosensor to detect glucose [126] (Figure 9e). Gox catalyzes the enzymatic reaction of glucose to release H+, resulting in a decrease in pH and the contraction of PAA chains. The orientation transition of LC molecules (from radial to bipolar) can be observed.

## 4. LC-Based Whispering Gallery Mode Microcavity Biosensing

WGM microcavities have attracted much attention due to their exceptionally high Q factor, small mode volume, and the ability to significantly increase the interaction between the light field and the matter in the cavity. As the optical microcavity of the WGM can confine resonant photons within the micrometre scale for an extensive period of time, photons can interact with the matter in the range of the resonant mode multiple times. This provides the WGM microcavity with an exceptionally high sensitivity, and has been applied to the detection of proteins [127], DNA [128], viruses [129], etc. LCs, as a branch of materials with fast response and high sensitivity, can be developed to generate WGM microresonators for biosensing detection on the basis of the LC amplification. Molecular binding events at the interface and external stimuli can trigger the orientation transition of LC molecules, and the change in the effective refractive index inside the WGM cavity is responsible for the shift in the resonant wavelength. Therefore, the resonant frequency of the WGM can be obtained by monitoring the transmission spectrum, the reflection spectrum, or the radiation spectrum of the microcavity, to achieve the biological detection. The LC-based WGM microcavity sensing provides a feasible solution for label-free optical biosensors with rapid response.

### 4.1. Classification and Preparation of WGM Microcavities

#### 4.1.1. Classification of WGM Microcavities

At present, the popular WGM microcavity structures include microspheres, microdisks, microrings, microtoroids, and microbubbles. The microsphere-based WGM resonator has a high Q factor and can accomplish the coupling of the evanescent field using a tapered optical fiber, which provides a promising application outlook with high sensitivity. Constraints in the microfabrication technology bring difficulties in producing smooth microdisks and microrings, resulting in a reduced Q factor and limited applications. The Q factors of general microdisk and microring cavities are approximately $10^{5}$. Lee et al. developed a wedge-shaped microdisk cavity with a Q factor as high as $10^{9}$ using the technique of chemical etching [130]. Armani et al. developed microtoroid cavities in order to address the issue of microdisk or microring cavities with low Q factors (caused by the surface roughness) [131]. The microtoroid cavity with a high Q factor was obtained by irradiating the microdisk cavity with a high-power laser to realize the reflow treatment. The structure of the microtoroid cavity was similar to those of the microdisk and the microring, but the boundary of the cavity was much more refined. Besides the high Q factor, the microtoroid cavity had a small mode volume, which was very conducive to building the highly sensitive sensor. Compared to other types of WGM microcavities, the hollow-cavity structure in the microbubble can inject the biomolecules into the cavity. This can allow the full interaction between the evanescent field and the biomolecules under test, and can prevent the signal crosstalk induced in the detection region and the coupling area. This, thus, provides an ideal platform for the highly-sensitive biological sensing.

#### 4.1.2. Fabrication of WGM Microcavities

Common microcavity materials include semiconductors (Si, ${\mathrm{SiO}}_{2}$, ${\mathrm{Si}}_{3}N_{4}$) and organic polymers (PDMS, PMMA). The cavities of different materials have various properties and application scenarios. The microsphere cavities are typically produced by melting and stretching the fiber end face using a hydrogen–oxygen flame or a carbon dioxide laser. The fiber end face forms a spherical shape with a smooth surface due to the surface tension. By modifying the parameters of the laser or the oxyhydrogen flame, and the diameter of the fiber, the size of the microspheres can be adjusted. Microdisk and microring cavities are fabricated mainly using advanced semiconductor lithography and micronano optical technologies. Microtoroid cavities are produced based on the conventional silicon-based wet etching technique, which is conducive to the integration of various optical devices on micro-silicon chips. Microbubble cavities are fabricated through the process of heating and pressure, and hollow microbubble cavities will be eventually produced by the surface tension of the silica. The laser output can be obtained by doping the polymer microcavity with fluorescent dyes [132]. The polymer material is low-cost and simple to process, but the polymer-based WGM cavity has a low Q factor and a short service life, which limits its applicability.

### 4.2. Applications of LC-Based Microcavities in Biosensing

Since Richtmyer et al. observed the WGM resonance phenomenon in a circular microcavity made of dielectric materials in 1939 [133], researchers have obtained further achievements in the areas of WGM. The combination of the fluidity of LCs and various optical microcavities can lead to the improvement of the performance. Due to the low surface-scattering loss, the Q factor of the internal WGM resonance can achieve a higher level. Meanwhile, because the refractive index of LCs is usually higher than those of most solutions, the condition of the total internal reflection can be easily satisfied to build a cavity.

**Figure 10:**
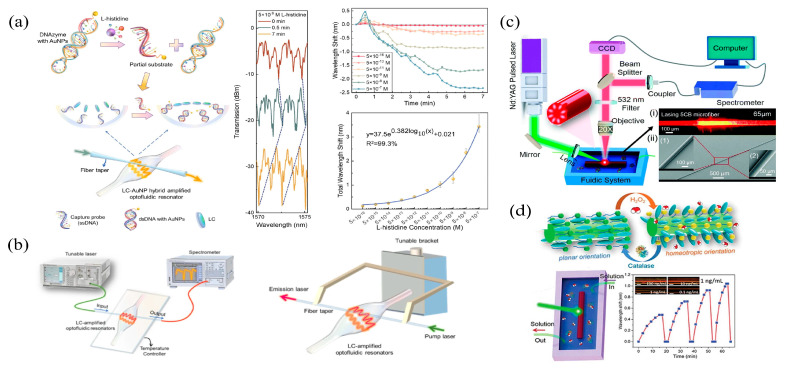
(**a**) Diagram of the DNAzyme optofluidic biosensor for L-histidine detection and the spectral response of the LC–AuNPs biosensor at different concentrations of L-histidine [134]; reproduced with permission from Elsevier. (**b**) Schematic of the optofluidic LC biosensor for protein assay detection [135]; reproduced with permission from Springer. (**c**) Illustration of the experimental setup for monitoring the concentration of lipase using the LC microfiber biosensor [136]; reproduced with permission from Royal Society of Chemistry. (**d**) Principle of the orientation transition of LCs, and the time response curve for the shift of WGM wavelength under various catalase concentrations [137]; reproduced with permission from Taylor & Francis.

Wang et al. developed a DNAzyme-based biosensor employing an LC–AuNPs hybrid amplified optofluidic resonator to realize the biodetection [134]. The microbubble resonator, with an outer diameter of 190 μm and a wall thickness of 4 μm, was obtained through the capillary stretching and expanding. This facilitated the evanescent field to enter the core and enhanced the interaction between the light and the matter (Figure 10a). L-histidine molecules can specifically cleave DANzyme. L-histidine molecules can specifically cleave DANzyme. With the release of the DNAzyme fragment (cleaved by L-histidine), it can hybridize with the capture probe (ssDNA) modified on the inner surface of the microbubble cavity to form the dsDNA. The orientation transition of LC molecules, from homeotropic to planar, was triggered. Due to the influence of the biomolecular hyperpolarization on the surface and the orientation transition of LC molecules, the ultrasensitive wavelength shift of the WGM spectroscopy can amplify and monitor minor changes in L-histidine molecules. In addition, AuNPs connected to partial substrates were able to increase the orientation transition of LCs and to boost the signal variation in the WGM laser spectrum. The spectral shift scaled with the increase of the L-histidine concentration, and the detection limit of the LC-based WGM optofluidic system reached the sub-femtomolar level (about $5\times 10^{-16}$ M). In addition, a high-sensitivity protein assay detection scheme using an LC-amplified optofluidic resonator was developed, taking advantage of high Q factor, strong evanescent field, and small mode volume of WGM cavities [135]. The protein adsorbed on the inner surface of the DMOAP-coated microcavity triggered the orientation transition of LC molecules, which was characterized by the wavelength shift of the WGM laser spectrum (Figure 10b). The sensitive wavelength shift in the spectrum was caused by the triple effect from the polarization of biomolecules, the orientation transition of LC molecules, and the WGM microcavity. Using BSA as the target protein, it was found that the total spectral shift scaled linearly as the BSA concentration increased, and the detection limit reached the femtomole-level (1.92 fM). Both the above experimental results provided a label-free, fast-response, and high-sensitivity detection solution for the analysis of analytes, which can be extended to sensitively detect other biomolecules.

Duan et al. proposed a WGM lasing-based LC soft-matter microfiber biosensor to implement the real-time, quantitative, and sensitive monitoring of lipase (Figure 10c), hydrogen peroxide, and catalase (Figure 10d) [136,137]. Since the cylindrically-structured LC microfiber resonator had the advantages of large sensing range and excellent potential for distributed detection of biochemical reactions, as well as outstanding flexibility and biocompatibility, 5CB microfibers were selected as components to fabricate optical microcavities in the biosensing experiment. Dodecaldehyde-doped 5CB was coated on the surface of polymethyl methacrylate (PMMA) to prepare functionalized 5CB microfibers. The spectral shift of the WGM laser was used as an effective indicator to detect the concentration of the target substance. Experiments on lipase achieved a detection limit of 0.01 μg ${\mathrm{mL}}^{-1}$, while experiments on hydrogen peroxide and catalase achieved detection limits of 0.26 μM and 1 ng ${\mathrm{mL}}^{-1}$, respectively. Eventually, an integrated distributed biochemical sensing platform based on soft-matter microfibers becomes feasible.

In addition to microbubble cavities, other optofluidic devices, e.g., liquid-core optical ring-cavities, can also employ LCs to realize the double-amplification biosensing. The small wall thickness of microbubble cavities and the liquid-core optical ring-cavities enable the external evanescent field to be fully coupled into the cavities [138,139]. As a result, when the biological solution enters the evanescent field region, the effective refractive index of the resonant mode varies more obviously, which can significantly improve the sensitivity against the substance under test. Although at present there have not been many LC-based optofluidic biosensors reported in practical applications, this novel sensing platform provides a new solution for the detection of biomolecules with a lower detection threshold than conventional techniques. This could bring a new research and development direction in the biosensing areas.

## 5. Conclusions and Outlooks

LC biosensors were developed based on the mechanism in which specific interactions between biotargets can disturb the orientation of LC molecules. This will lead to the change (in the color and the brightness) of visible optical images or the shift of the laser spectrum. Compared to traditional biosensing schemes, LC biosensors has the advantages of low cost, quick response, and simple operation, and it has become a leading option in the field of biosensors. This review comprehensively discussed the prosperous development and the significant progress in the LC biosensing technologies. LC sensors provide excellent detection performance with different structures, including platforms fabricated using conventional techniques, innovative microfluidic technologies (Table 1), and WGM optical microcavities (Table 2). Traditional LC biosensors have limitations such as low sensitivity and manual errors, despite their simple structure and easy operation. Therefore, the combination of the microfluidic technology and LCs can circumvent the aforementioned issues while keeping the merits of low cost and rapid response. Moreover, LC-based WGM optofluidic sensors have great application potential in the areas of biology and chemistry, and represent the state of the art of modern biosensing technologies.

Although LC sensors possess great application potentials, some issues need to be addressed in order to achieve their further commercialization. For LC biosensors using gold nanoparticles to amplify the signal, the experimental cost has been raised. Some metal materials can be dispersed in solvents as inorganic nanocores to form nanoparticles, which can be used as alternatives to AuNPs to amplify the signal [73,140]. The structure of microchannels and the impact of the phase viscosity and flow rate (of the liquid) on the formation of LC orientation based on the microfluidic technology need to be further investigated, so that the LC orientation transition process can be optimized. Fabrications of microfluidic chips can be modularized using the machine learning technologies [93,141]. Future research can also involve integrating WGM resonator, light source, and detector onto a single photonic chip to provide a cheap, portable, stable, and multifunctional LC biosensing platform [142]. Currently, the theories of biosensors based on WGM microcavities are relatively mature, but there are still a number of challenging issues to be resolved before the commercialization, e.g., long-term stability, noise suppression, surface functionalization, multiplexing technology, Fano resonance combination, and electrical signal enhancement [143]. 

## Figures and Tables

**Table 1:** Summary of different geometries of LC biosensors used in detecting biotargets.

Geometries of LC Biosensors	Fabrication Principle	Type of LC	Target	Detection Method	Detection Limit	Ref.
LC–solid interface	Glass-substrate	5CB	Alpha-synuclein	POM	50 nM	[72]
		5CB	Tetracycline	POM	0.5 pM	[74]
		5CB	Glycoprotein-120	POM	0.2 μg ${\mathrm{mL}}^{-1}$	[75]
		5CB	Amoxicillin	POM	3.5 nM	[77]
		E7	BSA	POM	$2.5\times 10^{-8}\;\mu$g ${\mathrm{mL}}^{-1}$	[78]
		E7	Cortisol	POM	$3\times 10^{-6}\;\mu$g ${\mathrm{mL}}^{-1}$	[78]
	Microfluidic	E44	BSA	POM	0.01 μg ${\mathrm{mL}}^{-1}$	[91]
		5CB	Anti-IgG	POM	0.02 mg ${\mathrm{mL}}^{-1}$	[92]
		5CB	Anti-IgG	POM	1 μg ${\mathrm{mL}}^{-1}$	[93]
LC–aqueous interface	Glass-substrate	5CB	Malathion	POM and gray values	0.465 nM	[97]
		5CB	Acetylcholine	POM and bright area coverage ratio	14.5 nM	[98]
		5CB	Catalase	POM and bright area coverage ratio	5.5 mU ${\mathrm{mL}}^{-1}$	[99]
		5CB	α-glucosidase (AGLU) inhibitors	POM and bright area coverage ratio	Acarbose (0.57 μM) Migliol (1.00 μM) Voglibose (0.01 μM)	[100]
	Microfluidic	5CB	Phospholipase	POM	100 nM	[102]
LC droplet	Glass-substrate	5CB	KB cancer cells	POM and bright field images	6000 cells ${\mathrm{mL}}^{-1}$	[108]
		5CB	HepG2 cells	POM and bright field images	1.0 ± 0.01 HepG2 cells μ$m^{-2}$	[109]
		5CB	SK-BR3 cancer cells	POM and bright field images	5000 cells ${\mathrm{mL}}^{-1}$	[110]
		5CB	BSA	Lasing spectra	1 pM	[111]
		5CB	Penicillinase	POM and lasing spectra	2μM	[112]
		5CB	AChE	POM and lasing spectra	Fenobucarb (0.1 pg) Dimethoate (1 pg ${\mathrm{mL}}^{-1}$)	[113]
	Microfluidic	5CB	Bile acids	POM and bright field images	Cholic acid (10 μM) Deoxycholic acid (1 μM)	[121]
		E7	$H_{2}O_{2}$	POM	$2.2\times 10^{-7}$μM	[123]
		E7	Ammonia	POM	$0.3\times 10^{-6}$ M	[124]
		E7	Antimicrobial peptides	POM	3.6 μM	[125]
		5CB	Glucose	POM	0.03 mM	[126]

**Table 2:** Summary of WGM microcavities of LC biosensors used in detecting biotargets.

Type of Microcavity	Type of LC	Target	Interaction Principle	Detection Limit	Ref.
Microbubbles made of silicon capillaries	5CB	L-histidine	Cleavage event of DNAzyme by the biological target leads to the shift of LC orientations	$5\times 10^{-16}$ M	[134]
Microbubbles made of silicon capillaries	5CB	BSA	BSA changes tilted LC molecular orientation	1.92 fM	[135]
PMMA microfibers functionalized with LC	5CB	Lipase	Enzymatic reaction between lipase and glycerol trioleate	0.01 μg ${\mathrm{mL}}^{-1}$	[136]
PMMA microfibers functionalized with LC	5CB	Hydrogen peroxide	Hydrogen peroxide contact with dodecanal-doped LC microfibers results in the shift of LC orientation	0.26 μM	[136]
PMMA microfibers functionalized with LC	5CB	Catalase	Degradation of hydrogen peroxide catalyzed by catalase	1 ng ${\mathrm{mL}}^{-1}$	[137]

## Data Availability

Not applicable.
